# *ITPR3* gene haplotype is associated with cervical squamous cell carcinoma risk in Taiwanese women

**DOI:** 10.18632/oncotarget.14341

**Published:** 2016-12-28

**Authors:** Yuh-Cheng Yang, Tzu-Yang Chang, Tze-Chien Chen, Wen-Shan Lin, Shih-Chuan Chang, Yann-Jinn Lee

**Affiliations:** ^1^ Department of Gynecology and Obstetrics, MacKay Memorial Hospital, Taipei City; ^2^ Department of Pediatric Endocrinology, MacKay Children's Hospital, Taipei City; ^3^ Department of Medical Research, MacKay Memorial Hospital, New Taipei City; ^4^ Department of Gynecology and Obstetrics, School of Medicine, College of Medicine, Taipei Medical University, Taipei City, Taiwan; ^5^ Departments of Gynecology and Obstetrics, Pediatrics, School of Medicine, College of Medicine, Taipei Medical University, Taipei City; ^6^ Institute of Biomedical Sciences Mackay Medical College, New Taipei City, Taiwan; ^7^ Department of Medicine, Mackay Medical College, New Taipei City, Taiwan

**Keywords:** cervical cancer, HPV, immunity, ITPR3, polymorphism

## Abstract

Host immunogenetic background plays an important role in human papillomavirus (HPV) infection and cervical cancer development. Inositol 1,4,5-triphosphate receptor type 3 (ITPR3) is essential for both immune activation and cancer pathogenesis. We aim to investigate if *ITPR3* genetic polymorphisms are associated with the risk of cervical cancer in Taiwanese women. *ITPR3 rs3748079 A/G* and *rs2229634 C/T* polymorphisms were genotyped in a hospital-based study of 462 women with cervical squamous cell carcinoma (CSCC) and 921 age-matched healthy control women. The presence and genotypes of HPV in CSCC was determined. No significant association of individual *ITPR3* variants were found among controls, CSCC, and HPV-16 positive CSCC. However, we found a significant association of haplotype *AT* between CSCC and controls (OR = 2.28, 95% CI 1.31–3.97, *P* = 2.83 × 10^−3^) and the OR increased further in CSCC patients infected with HPV-16 (OR = 2.89, 95% CI 1.55–5.37, *P* = 4.54 × 10^−4^). The linkage disequilibrium analysis demonstrated that *ITPR3* association with CSCC was independent of *HLA-DRB1* alleles. In conclusion, these findings suggest that *AT* haplotype in the *ITPR3* gene may serve as a potential marker for genetic susceptibility to CSCC.

## INTRODUCTION

Cervical cancer is the fourth most common cancer in women worldwide. In Taiwan, cervical cancer also poses a major public health concern, with nearly 2700 women were diagnosed with cervical cancer each year [1]. Infection with oncogenic human papillomavirus (HPV) is necessary for the development of cervical cancer [2]. However, the majority of women infected with HPV do not progress into cervical cancer, suggesting that other factors are required for the cancer to develop. A potential cofactor is the individual immune system, which may play a pivotal role in viral clearance and involve in the cervical cancer development.

The inositol 1,4,5-triphosphate receptor type 3 (ITPR3), an intracellular Ca^2+^ release channel on the endoplasmic reticulum membrane, is responsive to the binding of a second messenger inositol 1,4,5-triphosphate (IP3) [3]. IP3 is phosphorylated to become IP4 by inositol 1,4,5-triphosphate 3-kinase C (ITPKC) and negatively regulates T-cell receptor signal transduction [4]. A study reported by our group revealed that a genetic variant *rs28493229* of the *ITPKC* gene is significantly associated with increased risk of cervical cancer in Taiwanese women [5]. ITPR3 has been shown to participate in induction of apoptosis in T lymphocytes and other types of cells [6–8]. The expression level of ITPR3 is associated with growth and aggressiveness of different types of tumors [9–12]. In addition, a number of studies have demonstrated that specific single nucleotide polymorphisms (SNPs) in the *ITPR3* gene were associated with susceptibility to various immune-mediated diseases, such as type 1 diabetes, systemic lupus erythematosus, rheumatoid arthritis, and Graves’ disease [13–15].

Because of the importance of ITPR3 to the immunity and development of immune-associated diseases, it is reasonable to speculate that *ITPR3* genetic variants might participate in the cancer pathogenesis. We therefore tested the hypothesis that specific *ITPR3* SNPs are associated with cervical cancer risk with an association study of 462 cervical squamous cell carcinoma (CSCC) patients and 921 healthy controls.

## RESULTS

A total of 84.7% detection rate for HPV DNA was found in 462 CSCC samples. The HPV type distributions were observed that HPV 16 was 63.5%, HPV 18 was 9.8%, and the remaining types were 26.7%.

The genotype and allele distributions of *ITPR3* SNPs in cases and controls were shown in Tables 1 and 2. The genotype frequencies of all SNPs in the control group did not deviate significantly from Hardy-Weinberg equilibrium (*P* > 0.05). We did not find any significant differences in the genotype and allele frequencies for the 2 *ITPR3* SNPs between CSCC patients and controls (Tables 1 and 2). Based on the HPV-16 positivity stratification analysis, the synergistic effect of HPV-16 infection and *ITPR3* polymorphisms on the risk of CSCC can be explored. The results showed that no significant difference of genotype and allele distributions was observed between women with HPV-16 positive CSCC and controls (Tables 1 and 2). Linkage disequilibrium (LD) analyses between the 2 SNPs revealed strong LDs in both controls (*D′* = 0.87) and patients (*D′* = 0.72).

**Table 1:** Genotype and allele frequencies of the *ITPR3 rs3748079 A/G* polymorphism in controls and in women with CSCC and those with HPV-16 positive CSCC*

	Controls (N = 921)	CSCC (N = 462)	HPV-16 positive CSCC (N = 245)	CSCC		HPV-16 positive CSCC	
	No. (%)	No. (%)	No. (%)	*P* value (χ^2^)	OR (95% CI)	*P* value (χ^2^)	OR (95% CI)
Genotype				0.34 (2.16)		0.77 (0.53)	
A/A	40 (4.3)	28 (6.1)	13 (5.3)		1.42 (0.86-2.33)		1.23 (0.65-2.35)
A/G	301 (32.7)	154 (33.3)	82 (33.5)		1.03 (0.81-1.31)		1.04 (0.77-1.40)
G/G	580 (63.0)	280 (60.6)	150 (61.2)		0.90 (0.72-1.14)		0.93 (0.69-1.24)
Allele				0.22 (1.53)		0.51 (0.43)	
A	381 (20.7)	210 (22.7)	108 (22.0)		1.13 (0.93-1.36)		1.08 (0.85-1.38)
G	1461 (79.3)	714 (77.3)	382 (78.0)		0.89 (0.73-1.07)		0.92 (0.72-1.17)

* ITPR3 = inositol 1,4,5-trisphosphate receptor type 3; CSCC = cervical squamous cell carcinoma; HPV = human papillomavirus; OR = odds ratio; CI = confidence interval.

**Table 2:** Genotype and allele frequencies of the *ITPR3 rs2229634 C/T* polymorphism in controls and in women with CSCC and those with HPV-16 positive CSCC*

	Controls (N = 921)	CSCC (N = 462)	HPV-16 positive CSCC (N = 245)	CSCC		HPV-16 positive CSCC	
	No. (%)	No. (%)	No. (%)	*P* value (χ^2^)	OR (95% CI)	*P* value (χ^2^)	OR (95% CI)
Genotype				0.23 (2.90)		0.19 (3.34)	
C/C	251 (27.3)	146 (31.6)	75 (30.6)		1.23 (0.97-1.57)		1.18 (0.87-1.60)
C/T	440 (47.8)	205 (44.4)	101 (41.2)		0.87 (0.70-1.09)		0.77 (0.58-1.02)
T/T	230 (24.9)	111 (24.0)	69 (28.2)		0.95 (0.73-1.23)		1.18 (0.86-1.62)
Allele				0.19 (1.73)		0.97 (0.00)	
C	942 (51.1)	497 (53.8)	251 (51.2)		1.11 (0.95-1.30)		1.00 (0.82-1.22)
T	900 (48.9)	427 (46.2)	239 (48.8)		0.90 (0.77-1.05)		1.00 (0.82-1.22)

* ITPR3 = inositol 1,4,5-trisphosphate receptor type 3; CSCC = cervical squamous cell carcinoma; HPV = human papillomavirus; OR = odds ratio; CI = confidence interval.

The inferred haplotypes constructed by *rs3748079 A/G* and *rs2229634 C/T* SNPs were shown in Table 3. The overall *P* values achieved statistical significances (*P* = 7.0 × 10^−3^ and 4.8 × 10^−3^ in the CSCC and HPV-16 positive CSCC groups, respectively). We found *GT* was the most frequent but was not associated with CSCC after Bonferroni corrections. However, the frequencies of *AT* haplotype increased significantly in both CSCC patients (OR = 2.28, 95% CI 1.31–3.97, *P* = 2.83 × 10^−3^) and HPV-16 positive CSCC patients (OR = 2.89, 95% CI 1.55–5.37, *P* = 4.54 × 10^−4^), which remained significant even after correction for multiple comparisons (*P_c_* = 0.01 and 1.81 × 10^−3^, respectively) (Table 3).

**Table 3:** Analysis of *ITPR3* haplotypes in controls and in women with CSCC and those with HPV-16 positive CSCC *

Haplotype	Controls (2N = 1842)	CSCC (2N = 924)	HPV-16 positive CSCC (2N = 490)	CSCC		HPV-16 positive CSCC	
	No. (%)	No. (%)	No. (%)	*P* value (χ^2^)	OR (95% CI)	*P* value (χ^2^)	OR (95% CI)
GT	875 (47.5)	400 (43.3)	221 (45.0)	0.04 (4.39)	0.84 (0.72-0.99)	0.34 (0.90)	0.91 (0.74-1.11)
GC	586 (31.8)	314 (34.0)	161 (32.9)	0.25 (1.32)	1.10 (0.93-1.30)	0.66 (0.19)	1.05 (0.85-1.30)
AC	357 (19.4)	183 (19.8)	90 (18.3)	0.79 (0.07)	1.03 (0.84-1.25)	0.61 (0.26)	0.94 (0.72-1.21)
AT	24 (1.3)	27 (2.9)	18 (3.7)	2.83 × 10^−3^ (8.91)	2.28 (1.31-3.97)	4.54 × 10^−4^ (12.3)	2.89 (1.55-5.37)

* Haplotype inferred using Haploview 4.2 program, based on the order of *rs3748079 A/G* and *rs2229634 C/T* polymorphisms.

ITPR3 = inositol 1,4,5-trisphosphate receptor type 3; CSCC = cervical squamous cell carcinoma; HPV = human papillomavirus; OR = odds ratio; CI = confidence interval.

*P* value for 4 haplotypes between all CSCC patients and controls: *P* = 7.0 × 10^−3^ (χ^2^ = 12.1, 3 df).

*P* value for 4 haplotypes between HPV-16 positive CSCC patients and controls: *P* = 4.8 × 10^−3^ (χ^2^ = 12.9, 3 df).

## DISCUSSION

In this study, we investigated the association of *ITPR3 rs3748079 A/G* and *rs2229634 C/T* SNPs and their haplotypes with the cervical cancer risk in Taiwanese women. The rationale for choosing these 2 variants is based on their potential functional implication in *ITPR3* gene. The *rs3748079* SNP, located in promoter region of *ITPR3* gene, has been found to upregulate *ITPR3* mRNA expression in cells having *A* allele [14]. Another variant, *rs2229634*, is a synonymous SNP situated in exon 20, which might affect mRNA stability and splicing [16]. We found that, individually, *rs3748079* and *rs2229634* SNPs were not associated with CSCC susceptibility. However, the haplotype *rs3748079 A-rs2229634 T* conferred a risk of CSCC patients and the risk increased further in CSCC patients infected with HPV-16. The limitations of our study include a selection bias existing in the study of retrospective design and scarcity of screened SNPs. Therefore, a large-scale prospective study that investigates other SNPs of *ITPR3* genes is needed to confirm our findings.

Ca^2+^ serves as a universal second messenger in virtually all cells. It plays a central role in regulating proliferation/activation and apoptosis of both immune and cancer cells [17, 18]. When cells are challenged with diverse extracellular stimuli, phospholipase C pathway is activated and results in generation of IP3, which binds to IP3 receptors and releases Ca^2+^ from the endoplasmic reticulum. IP3 receptors are a principal route of Ca^2+^ flux and this makes them an excellent candidate for immune- and cancer-associated studies. An investigation performed by Khan et al. found that increased ITPR3 may be causally related to lymphocyte apoptosis [6]. Other reports showed that ITPR1 and ITPR3 are critical for T-cell activation because of their involvement in IL-2 and IFN-γ production [19, 20]. In addition, a few studies found that overexpression of ITPR3 in breast and colon cancer cells decreased apoptosis, while knockdown of the receptor enhanced apoptosis [9, 10]. Cancer cells also exploit various kinds of proto-oncogenes and tumor-suppressors to modulate the expression and function of IP3 receptors in their favor [21]. These findings strongly support a potential role for ITPR3 in carcinogenesis. However, the associations between individual *ITPR3* gene polymorphism and CSCC risk have not been discovered in our study.

A haplotype is a set of DNA variations along a chromosome, which tends to be inherited together. A few studies have found that haplotypes may contribute to a phenotype through the combined effects of multiple individual SNPs on promoter activity or protein function [22, 23]. The hypothesis that haplotypes are more important than SNPs has been supported by both simulated and empirical data. A simulation study reported by Akey et al. showed that haplotypes can significantly improve the power of an association test [24]. They also used published data from the hereditary hemochromatosis disease region to validate their findings. Another study conducted by Zhang et al. used coalescent simulations to infer haplotype frequencies and quantitatively assess the power in both case-control and case-parental control designs [25]. They found that haplotype-based analysis is always more powerful than marker-by-marker analysis. Fallin et al. applied expectation-maximization algorithm estimated haplotype frequencies to investigate associations between apolipoprotein E SNPs and Alzheimer's disease [26]. They showed that significant associations were obtained for haplotypes that were not identified using individual SNPs. These results clearly provide examples that haplotype analysis is more informative than association studies based on a single marker.

To the best of our knowledge, this study is the first to explore the association between *ITPR3* gene haplotypes and cervical cancer. The *AT* haplotype was found to be associated with risk of women with CSCC and HPV-16 positive CSCC even though the *rs3748079 A/G* or *rs2229634 C/T* polymorphism alone was not. Thus, we speculate that the haplotype *AT* may have a role in *ITPR3* gene regulation and function and involve in a mechanism that determines the outcome of HPV infection and the subsequent development of CSCC. There are several possible explanations for these findings. The interaction of multiple SNPs within a haplotype may ultimately produce the biologic phenotype and become a powerful determinant of disease susceptibility. Another possibility is that the specific *AT* haplotype found in this study is in LD with the class II *HLA* loci.

Because *ITPR3* locates at about 500kb centrometric to the class II *HLA* genes DR and DQ, which are found to associate with cervical cancer [27, 28], it is important to clarify whether association between *ITPR3 AT* haplotype and CSCC is dependent on LD with class II *HLA* genes. The LD between *ITPR3* and *HLA-DRB1* alleles was analyzed in the subgroup of 333 CSCC patients and 241 controls using the PyPop software [29]. In this subgroup, we did not find any significant associations between *HLA-DRB1* alleles and CSCC. Only weak LD was identified between *ITPR3* and *HLA-DRB1* alleles in the control subset (*D′* = 0.31 and 0.34, respectively). These lines of evidence suggest that it is unlikely that the *ITPR3 AT* haplotype association with CSCC is dependent on *HLA-DRB1* locus. However, we are unable to exclude the LD effect of *HLA-DQB1* alleles or *DRB1-DQB1* haplotypes on the association of *ITPR3 AT* haplotype with CSCC.

In summary, our study demonstrates that the *AT* haplotype in *ITPR3* gene is markedly associated with CSCC risk in Taiwanese women. Additionally, women carrying the *AT* haplotype and harboring HPV-16 infection enhance susceptibility to CSCC. Further investigations are warranted to elucidate the specific role of ITPR3 molecule on the etiology of CSCC.

## PATIENTS AND METHODS

### Study subjects

The study included 462 patients with CSCC (mean ± SD age at diagnosis: 53.2 ± 13.1 years) residing in northern Taiwan. The diagnosis of CSCC was confirmed by histological examinations of tissues from biopsies or resected specimens. The control group consisted of 921 sex- and age-matched healthy subjects (mean ± SD age at sampling: 52.4 ± 12.1 years) who were enrolled from women attending the routine Pap screening and with normal Pap smear and no previous history of cervical dysplasia. The cases and controls were all genetically unrelated Taiwanese women. Informed written consent was obtained from all participants for use of their surgical resections or cervical scrapings for the study. The study protocol conformed to the ethical guidelines of the 1964 Declaration of Helsinki and was approved by the Institutional Review Board of Mackay Memorial Hospital.

### DNA extraction

Formalin-fixed, paraffin-embedded tissue blocks from CSCC patients were sectioned and dewaxed, and genomic DNA was then extracted using the Qiagen DNeasy Tissue Kit (Qiagen, Valencia, CA) according to the manufacturer's protocol. Genomic DNA of controls was extracted from cervical scrapings using Qiagen DNA extraction kit.

### HPV Detection and Typing

The detection of HPV and genotyping on 462 cervical DNA samples were performed by polymerase chain reaction (PCR). A pair of degenerate primers, GP6+/MY11, designed according to the highly conserved domain, was used to amplify a fragment of approximately 192 bp in the L1 region of the HPV genome [30, 31]. The PCR product was then sequenced on an automated sequencer (ABI 377, Applied Biosystems, Foster City, CA) to determine the HPV genotype. Since stratifications based on HPV types in controls were not performed in this study, no HPV DNA testing was done for the 921 control subjects.

### *ITPR3* genotyping

*ITPR3 rs3748079 A/G* and *rs2229634 C/T* polymorphisms were genotyped. They were determined using the Pre-Developed TaqMan Allelic Discrimination Assay (Applied Biosystems, Foster City, CA). Briefly, polymerase chain reactions (PCR) were carried out in a 96-well GeneAmp PCR System 9700 (Applied Biosystems) with mixes consisting of 10 ng of genomic DNA, 5 μl of TaqMan Universal PCR Master Mix, 0.5 μl of 20× Assay Mix, and ddH_2_O to a final volume of 10 μl. Thermal cycle conditions were as follows: denaturation at 95°C for 10 min, followed by 40 cycles of denaturation at 92°C for 15 sec, and annealing and extension at 60°C for 1 min. After PCR, the TaqMan assay plates were transferred to the ABI PRISM 7000 Sequence Detection System (Applied Biosystems) where the endpoint fluorescence intensity in each well of the plate was read. The allele specific fluorescence data from each plate were analyzed using the SDS v.1.1 software (Applied Biosystems) to automatically determine the genotype of each sample.

### Statistical analysis

Genotype and allele frequencies of the *ITPR3 rs3748079 A/G* and *rs2229634 C/T* SNPs were determined by direct counting. The Hardy-Weinberg equilibrium was assessed for each SNP in both the control and case groups by chi-square analysis. Linkage analysis and the frequencies of *ITPR3* haplotypes in controls and cases were estimated using the Haploview 4.2 program [32].

Statistical differences in the genotype, allele, and haplotype distributions between controls and cases were performed by chi-square test with Yates’ correction or Fisher's exact test (when the number of subjects in a cell was <5). Odds ratios (OR) and 95% confidence intervals (CI) were also calculated. Bonferroni correction for multiple testing was applied by multiplying *P* value with the number of comparisons performed. *P_c_* values of less than 0.05 (2-tailed) were considered to be statistically significant.

Prior to the study, statistical power to detect effects of the *ITPR3* SNPs on susceptibility to CSCC was calculated using the Quanto Ver. 1.1 software (Department of Preventive Medicine, University of Southern California, CA, USA). We designed the study to have a power >95% to determine a relative risk of 1.4 for the genotype of each SNP at a significance level of 0.05, with an estimated prevalence of CSCC of 360/100,000 [33].
